# Real Medicine

**DOI:** 10.1007/s11606-026-10644-8

**Published:** 2026-07-21

**Authors:** Tim Mercer

**Affiliations:** Division of Global Health, The Departments of Population Health and Internal Medicine, The University of Texas at Austin Dell Medical School; Central Health, Travis County Hospital District, Austin, TX USA

Howard first came to see me at Sunrise, the “Weirdest Little Church in Texas,” or so their sign said. He was towering in green army fatigues and a hefty backpack. Powder blue eyes penetrated deep from a pale and delicate face framed by a prominent jawline. His dusty orange hair was pulled back in a ponytail, and he spoke slow, with a Texas drawl.

Sunrise served coffee and breakfast to their neighbors experiencing homelessness and were the mailing address for several hundred. Most importantly, they were a trusted haven in the city for our patients who had little reason to trust anyone. We partnered with them early to establish a weekly mobile clinic on site. I was new in town, fresh out of my residency at a large, academic medical center, and then two years in Kenya attending on the overflowing medical wards of one of the busiest public hospitals in the country. Compared to managing heart failure from rheumatic heart disease or treating extrapulmonary tuberculosis, I did not feel like I was doing much for Howard. I mostly gave him naproxen for his back pain. To be honest, I wasn’t sure if this was even real medicine.

Howard kept coming back after I transitioned to a more traditional clinic not far away, still serving people experiencing homelessness. Over time, we got him an MRI and figured out he had lumbar spinal stenosis and severe degenerative disc disease. He saw a neurosurgeon and got some spinal injections. His symptoms ebbed and flowed but generally improved. Then, other problems started creeping up. Half his body would go numb. But it wasn’t a stroke. He would have headaches or vomiting. He developed seizures, often describing that his whole body would just “shut down.” Pain would radiate between body parts that were not anatomically connected. It was distressing. I listened intently, clarifying details when I could. I almost never knew what to make of this. Sometimes there were new diagnostic tests to order, treatment strategies to try, or referrals to make; sometimes there was not. Our visits would, ultimately, reach a culmination, we would make a follow-up plan, and he would come back. Again, I still wasn’t sure if this was real medicine.

At some point, the name in our electronic health record changed to Holly. It was kind of a non-event. She was open about it and nonchalant. I could not shake from my mind the towering Howard in green army fatigues with a huge pack who had first come to see me in my church-based mobile clinic. Now, she was in cut-off jean shorts and a black tank top. A tattoo of a whimsical twirling elephant in a red tutu danced on her arm. It was beautiful and my gaze was drawn to it. Had it been there all along? I struggled with her name and pronouns for the better part of a year. It was so embarrassing. She was always forgiving and gracious. Eventually, I got it right.

Holly had been enrolled in our integrated care team to address medical needs, mental health, addiction, and housing. She was receiving gender-affirming care from another community-based clinic, but sometimes I would bridge her prescriptions as needed. She would laugh and proudly show me her negative STI panel that she got monthly when I worked up the courage to ask her about her sexual risk. She was doing okay.

Throughout all this, I learned Holly was using a lot of methamphetamines. Maybe this explained some of her symptoms? We developed enough trust over the years that we could talk openly about it. Sometimes I would work it in during our discourse about her symptomatology. Sometimes she would agree, and I would have my way out. Other times she would shrug me off, and again, with those penetrating eyes, probe me for an alternative diagnosis. After the pandemic, our conversations about how fentanyl was poisoning the local drug supply got real. She had lost several friends to overdoses. I had lost some patients too. We grieved together during brief moments of silence that our conversations would inevitably bring us to. She said she would not risk overdosing like her friends. She had seen too many people die on the street. Eventually, many of her symptoms subsided and she was now in recovery. I often reflected on our visits, and was grateful, but realized I had mostly just listened. Was this real medicine?

Seven years after I met Howard, and continued to care for Holly, she came to my clinic for what was to be our final visit. I was staying in the same city but taking on a new role within our local safety net health system. I sat, laptop on my knees, on the black swiveling stool of every typical clinic exam room. I relayed the news to Holly, and she sat back, unblinking, the sparkling black and red elephant dancing on her folded arm. Then, she leaned forward quickly, suddenly towering again, and with a mix of anger and love, said in her slow, Texas drawl, “I promise you, I will never come to see another doctor ever again.” Her light blue eyes penetrated as deep as they ever had. Then, she sat back and her eyes welled with tears. So did mine. I shifted and swiveled in my stool, eyes darting from her gaze to the dancing elephant, and then down to the floor. I murmured some platitudes. As usual, she made it easy for me and we concluded our visit, just as we had done all those times before. She was graceful, and grateful, and felt heard and cared for. I told her I would ensure she was cared for by one of my colleagues I trusted. She shrugged, and smiled as if to reassure me, when I was supposed to be the one reassuring her. We hugged, and she told me she loved me. I told her I loved her too. This was, indeed, real medicine all along.

